# GloEC: a hierarchical-aware global model for predicting enzyme function

**DOI:** 10.1093/bib/bbae365

**Published:** 2024-07-29

**Authors:** Yiran Huang, Yufu Lin, Wei Lan, Cuiyu Huang, Cheng Zhong

**Affiliations:** School of Computer, Electronics and Information, Guangxi University, Nanning 530004, China; Key Laboratory of Parallel, Distributed and Intelligent Computing in Guangxi Universities and Colleges, Guangxi University, Nanning 530004, China; Guangxi Key Laboratory of Multimedia Communications and Network Technology, Guangxi University, Nanning 530004, China; School of Computer, Electronics and Information, Guangxi University, Nanning 530004, China; School of Computer, Electronics and Information, Guangxi University, Nanning 530004, China; Key Laboratory of Parallel, Distributed and Intelligent Computing in Guangxi Universities and Colleges, Guangxi University, Nanning 530004, China; Guangxi Key Laboratory of Multimedia Communications and Network Technology, Guangxi University, Nanning 530004, China; College of Chemistry, Tianjin Key Laboratory of Biosensing and Molecular Recognition, Nankai University, Tianjin 300071, China; School of Computer, Electronics and Information, Guangxi University, Nanning 530004, China; Key Laboratory of Parallel, Distributed and Intelligent Computing in Guangxi Universities and Colleges, Guangxi University, Nanning 530004, China; Guangxi Key Laboratory of Multimedia Communications and Network Technology, Guangxi University, Nanning 530004, China

**Keywords:** enzyme, Enzyme Commission number, Graph Convolutional Network, end-to-end

## Abstract

The annotation of enzyme function is a fundamental challenge in industrial biotechnology and pathologies. Numerous computational methods have been proposed to predict enzyme function by annotating enzyme labels with Enzyme Commission number. However, the existing methods face difficulties in modelling the hierarchical structure of enzyme label in a global view. Moreover, they haven’t gone entirely to leverage the mutual interactions between different levels of enzyme label. In this paper, we formulate the hierarchy of enzyme label as a directed enzyme graph and propose a hierarchy-GCN (Graph Convolutional Network) encoder to globally model enzyme label dependency on the enzyme graph. Based on the enzyme hierarchy encoder, we develop an end-to-end hierarchical-aware global model named GloEC to predict enzyme function. GloEC learns hierarchical-aware enzyme label embeddings via the hierarchy-GCN encoder and conducts deductive fusion of label-aware enzyme features to predict enzyme labels. Meanwhile, our hierarchy-GCN encoder is designed to bidirectionally compute to investigate the enzyme label correlation information in both bottom-up and top-down manners, which has not been explored in enzyme function prediction. Comparative experiments on three benchmark datasets show that GloEC achieves better predictive performance as compared to the existing methods. The case studies also demonstrate that GloEC is capable of effectively predicting the function of isoenzyme. GloEC is available at: https://github.com/hyr0771/GloEC.

## Introduction

Enzyme is one of the important proteins in living organisms and plays a catalytic role in various processes of life activities, including metabolism, nutrition, and energy conversion [1, 2]. It is thus of great significance to identify the function of protein enzymes expressed by genes [3–5]. According to the Swiss-Prot database [6] (as of June 2023), 274,340 out of 570 420 manually annotated proteins are enzymes. Such numerous enzymes are commonly classified by the Enzyme Commission (EC) system [7]. The EC system annotates the function of an enzyme with a four-digit EC number. Some machine learning methods have been proposed to classify the function of enzymes through precisely predicting the EC numbers [8]. For example, Tao *et al* [9] used artificial neural networks to capture protein sequences and biological prior features to classify proteins into seven distinct enzyme major classes. Concu *et al*. [10] proposed a quantitative structure–activity relationship method QSAR to divide proteins into seven enzymes and subclasses. Based on the contrast learning architecture, CLEAN [11] takes protein sequence as input and produces a list of EC numbers scored by comparing Euclidean distances between sequences.

Recently, people seek to apply deep learning predictor to encode protein [12] and identify enzyme EC numbers. Based on the rational, the deep learning methods for predicting enzyme function could be categorized into two groups: local approaches and global approaches.

The local approaches adopt level-by-level strategy to build hierarchy classification model to predict each EC level label for enzyme. DEEPre [13] constructs a convolutional neural network (CNN) model to identify the protein sequence as enzyme or non-enzyme, a model to classify the first EC level of enzyme and six models to classify the second EC level of enzyme. Similar to DEEPre, DeepEC [14] implements three CNN models to identify the enzyme EC numbers. The first CNN of DeepEC classifies whether the protein sequence is an enzyme or not. The second and third CNN determine the third and fourth EC levels of enzyme respectively. HECNet [15] employs Siamese and Triplet Networks [16, 17] to perform enzyme classification, in which 5 models are trained for determining the first EC level of enzyme and 13 models are trained for determining the second to fourth level of enzyme. Although constructing hierarchy classification model level-by-level can precisely capture level-specific features for classifying enzymes, they fail to model the enzyme label space in a global view.

To alleviate the abovementioned limitation, the global approaches treat the enzyme function prediction as a flat multi-label classification task and globally utilize one single classifier for all enzyme classes at the target level. COFACTOR [18] aligns the target protein structure with the template library and assigns the EC number of the most similar template enzyme to the target protein. ProteInfer [19] implements a deep extended convolutional architecture for predicting enzyme function, in which enzyme sequence is gradually expanded through a series of convolution within the residual block and continuous filters. Strodthoff *et al.* [20] proposed a self-supervised learning model UDSMProt that is pre-trained with the unlabeled protein sequence of Swiss-Prot database [6] to globally implement enzyme class prediction. Similarly, DAttProt [21] pre-trains transformer encoders to find and represent the correlations of protein sequences from the Swiss-Prot database, and employs multi-scale convolutions to extract the global features of the encoded protein sequences for predicting enzyme class. A synopsis on the prediction methods for enzyme EC number is listed in Table 1.

**Table 1:** A synopsis on the prediction methods for enzyme EC number.

Name	Description	Reference
CLEAN	The protein sequences are used as input to generate a list of EC numbers with scores by comparing the Euclidean distances between sequences.	[11]
DEEPre	A hierarchical strategy is adopted to construct a hierarchical classification CNN model to predict EC level label of enzyme.	[13]
DeepEC	Three CNN models are utilized for the recognition of enzyme EC numbers.	[14]
HECNet	Siamese and Triplet Networks are employed to train multiple models for predicting enzymes of various EC levels.	[15–17]
COFACTOR	A single classifier is used to match the target protein structure with template enzyme and assign the target protein with the EC number of the most similar template enzyme.	[18]
ProteInfer	A deep dilated convolutional architecture is designed for enzyme function prediction, allowing the top residual layers of the network to build up a representation of high-order protein features.	[19]
UDSMProt	Self-supervised learning is used to pre-train the unlabeled protein sequences for predicting enzyme EC number.	[20]
DAttProt	Transformer encoders are used to pre-train protein sequences, and multi-scale convolutions are employed to extract global features to predict enzyme functions.	[21]
ECRECer	An extreme multi-label classifier is used for EC number prediction, and a greedy strategy is adopted to ensemble and fine-tune the final model.	[22]
PredictEFC	Random forests are chosen as the base classification algorithm and the classifier is constructed using random k-label sets for classifying enzyme functions.	[23, 24]

It’s worth noting that the prediction of higher enzyme EC level relies on the identification of the lower enzyme EC level. The label-related information between different enzyme EC levels thus could enable us to accurately identify enzyme EC numbers. Although both local and global approaches have achieved success in capturing structure information of enzyme label for enzyme function prediction, the holistic label-correlation hierarchy information of different enzyme EC levels has not yet been fully investigated in enzyme function predictions.

In order to tackle this problem, we construct a hierarchy-aware global model called GloEC for predicting enzyme function using Graph Convolutional Network (GCN). It comprises a sequence encoder for extracting enzyme sequence features and a hierarchy encoder for modeling hierarchical enzyme label correlations. GloEC offers the following advantages over its counterparts:

(1) GloEC globally formulates the taxonomic hierarchy of enzyme as a directed enzyme graph and combines the enzyme graph to develop a hierarchy-GCN encoder to model the hierarchical dependency of enzyme labels, thus extracting fine-grained label-correlation hierarchy information of enzymes.
(2) The hierarchy-GCN encoder is bidirectionally computed. This can enable us to capture the label correlation information of enzymes in both bottom-up and top-down manners, which has not been explored for enzyme function classification.

To evaluate the robustness and accuracy of GloEC, we conduct comparative experiments on three benchmark datasets. The results on the benchmark datasets show that GloEC achieves better prediction performance than the state-of-the-art methods. Furthermore, study cases also confirm that GloEC is capable of accurately predicting isoenzyme functions. These results demonstrate that GloEC is a feasible and effective model for identifying enzyme functions.

## Materials and methods

### Dataset

In this work, we collated three benchmark datasets for study. First, we collected all the enzymes from the Swiss-Prot database [6], obtaining a total of 274 340 enzymes (the highest level up to level 4) as of June 2023. To obtain a high-quality dataset, the enzyme data are cleaned by using the following steps:

(1) To ensure that the data is non-redundant, the CD-HIT [25] tool is used to eliminate the enzyme sequences with a similarity threshold of 50%. When the similarity between sequences exceeds 50%, it is generally considered that they have similar functionalities and structures [26, 27]. Removing such redundant data can help to reduce bias in model development and ensure complete sequence coverage.
(2) To ensure uniqueness and correctness, the enzyme sequences associated with multiple EC numbers were removed. Enzyme EC numbers provide crucial information regarding their functionalities and catalytic activities. Enzymes with multiple EC numbers indicate their involvement in various functions; however, they are beyond the scope of discussion in this work.
(3) To ensure complete annotations for model training, the enzyme sequences with fewer than three EC number levels were removed. Higher-level EC numbers provide more specific functional descriptions. Enzyme sequences with lower-level EC numbers tend to contain more noise or redundant information, thus interfering with the model’s learning process.
(4) Following [15], in order to include as much samples as possible in each enzyme class for training, the enzyme sequences within the enzyme class containing less than 10 enzymes were removed.

After the above three steps, 36 854 enzyme sequences were retained, including 1635 EC numbers. Then we screened the enzymes included in the database from May 2022 to June 2023 as the test dataset. Since this test dataset contains 144 EC numbers and has 432 enzyme sequence samples, we call it New-432 dataset. The remaining data, including 36,422 enzyme sequences and 1635 EC numbers, were used as a basic training dataset.

Besides the benchmark dataset New-432, we also used the COFACTOR dataset retrieved from [28] for cross-dataset validation. The COFACTOR dataset satisfies that the pair-wise sequence similarity is less than 30% and no self-BLAST hit exists. This ensures no homologous enzymes in the dataset [13]. To avoid overlaps, we removed the same samples of the COFACTOR dataset and the basic training set, and reduced the number of the enzyme sequence samples from 318 to 237. This updated COFACTOR dataset is hereinafter referred to as the COFACTOR-237 dataset.

In addition to the baseline datasets New-432 dataset and COFACTOR-237, we also collected all isoenzymes from Swiss-port (as of June 2023) to test the ability of the proposed model to predict the function of enzyme subtypes. First, the enzyme entries that are more than 50% similar to the basic training dataset were removed in this dataset. Moreover, the enzyme sequences with multiple EC numbers and the enzymes whose EC numbers are not included in the basic training dataset were also removed. Finally, we obtained a dataset containing 237 enzyme EC numbers and 564 enzyme sequences, which is called Isoenzyme dataset.

The carbohydrate esterase family is built upon sequence homology, which exhibit multiple functions due to minor differences in sequences [29]. In order to learn the classification performance of the proposed model on enzymes from the same family, we also collected the carbohydrate esterase family from the TrEMBL database [5], which is called the Carbohydrate esterase dataset. This enzyme family contain 354 enzyme samples and these samples are classified into seven distinct EC numbers, with the allocation being determined by their specific catalyzed reaction types and substrate specificity.

### Model

#### Problem description

In the enzyme function prediction problem, a predefined taxonomic hierarchy is used to organize the enzyme label space. The taxonomic hierarchy mainly includes the directed acyclic graph (DAG) structure and the tree-like structure [30]. The DAG structure can be transformed into tree-like structure through distinguishing the label node to a single-path node. Hence the taxonomic hierarchy of enzyme label space can be reducible to a tree-like structure [30].

**Figure 1:**
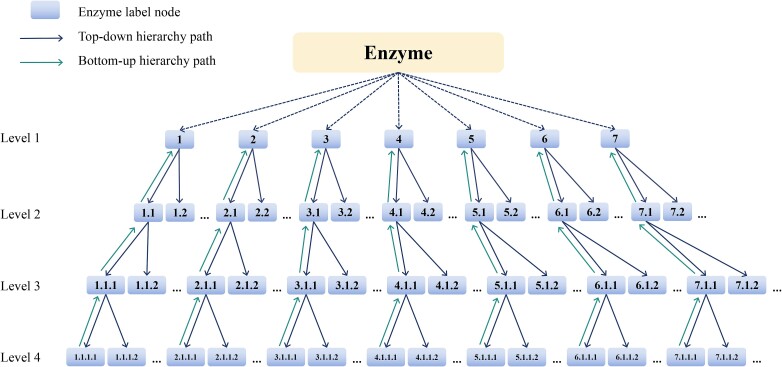
An example of enzyme taxonomic hierarchy graph. There are four enzyme label levels in the graph and each node is an enzyme EC label. The dependencies of each label are represented by arrows.

As shown in Fig. 1, we use a directed enzyme graph $G=(V,\overrightarrow{E},\overleftarrow{E})$ to formulate the taxonomic hierarchy of enzyme label space. In $G=(V,\overrightarrow{E},\overleftarrow{E})$, *V =* {*v*_1_, *v*_2_, *…v_i_*,*…*, *v_C_*} refers to the node set of enzyme labels in *G* and *C* denotes the number of enzyme label nodes. $\overrightarrow{E}=\left\{\left({v}_i,{v}_j\right)\left|{v}_i\in V,{v}_j\in child\left({v}_i\right)\right.\right\}$ is the top-down hierarchy path in *G* and child(*v_i_*) denotes the set of children for the enzyme label *v_i_*. $\overleftarrow{E}=\left\{\left({v}_j,{v}_i\right)\left|{v}_i\in V,{v}_j\in child\left({v}_i\right)\right.\right\}$ is the bottom-up hierarchy path. We call this tree-like directed graph $G=(V,\overrightarrow{E},\overleftarrow{E})$ as enzyme taxonomic hierarchy graph.

Given an enzyme sequence *x* and an enzyme taxonomic hierarchy graph $G=(V,\overrightarrow{E},\overleftarrow{E})$, the goal of enzyme level classification is to classify *x* to an enzyme label ${v}_i\in V$ in *G*.

**Figure 2:**
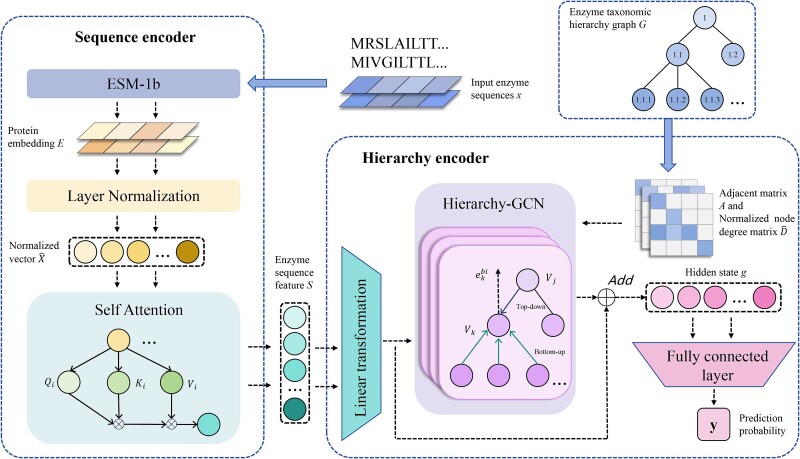
The overall structure of our global classification model GloEC. GloEC consists of two components: the sequence encoder and the hierarchy encoder. The sequence encoder extracts the enzyme features from the input enzyme sequence *x* by self-attention mechanism. The hierarchy encoder builds a hierarchy-GCN encoder with three-layer GCN network to aggregate the taxonomic hierarchy of the extracted enzyme features with the known enzyme taxonomic hierarchy graph *G*, and produce the prediction probability of the enzyme label nodes of *G* for the input enzyme sequence *x*.

In order to solve this problem, as can be seen in Fig. 2, our model GloEC consists of a sequence encoder and a hierarchy encoder. The sequence encoder extracts the features of the given enzyme sequence. The hierarchy encoder utilizes GCN to encode the extracted sequence features with the known enzyme taxonomic hierarchy graph to predict enzyme label for the given sequence. The following section will discuss our model in detail.

#### Sequence encoder

The first step of our model is to extract the features of the given protein sequence using sequence encoder. ESM-1b [31] is a protein language model, which is capable of encoding protein embeddings with semantically rich information. In our sequence encoder, we first use ESM-1b to encode the given enzyme sequence *x* into the protein embedding $E\in{\mathbb{R}}^{1280}$. Then a layer normalization module [32] is adopted to normalize the feature vector of the protein embedding *E* to reduce the dependence of neural network on batch size. Finally, self-attention mechanism [33] is utilized to extract the entire enzyme sequence information in the normalized feature vector from different representation subspaces.

Specifically, for the protein embedding *E*, we subtract the mean vector of *E* from *E* to produce the vector *U*. We then divide *U* by the standard deviation vector of *E* to obtain the normalized vector $\hat{X}\in{\mathbb{R}}^{d_n}$, ${d}_n$ is the output dimension of the layer normalization module.

Then self-attention mechanism takes the normalized vector $\hat{X}$ as input to capture the enzyme sequence features. Given *h* groups of trainable matrices $\boldsymbol{W}_j^Q\in{\mathbb{R}}^{d_n\times{d}_k},\boldsymbol{W}_j^K\in{\mathbb{R}}^{d_n\times{d}_k},\boldsymbol{W}_j^V\in{\mathbb{R}}^{d_n\times{d}_k},j\in h$, we use formula (1) to calculate the ${Q}_j\in{\mathbb{R}}^{d_k}$ (Query), ${K}_j\in{\mathbb{R}}^{d_k}$(Key) and ${V}_j\in{\mathbb{R}}^{d_k}$ (Value) matrices of $\hat{X}$ for the attention module in self-attention mechanism. *d_k_* is the output dimension of the self-attention module.

Based on the matrices ${Q}_j$, ${K}_j$ and ${V}_j$, we first use formula (2) to calculate the attention vector ${head}_j\in{\mathbb{R}}^{d_k}$ for the *j*th head of the attention module and then concatenate all attention vectors together to produce an enzyme sequence feature matrix $S\in{\mathbb{R}}^{d_k\times h}$ for the hierarchy encoder:


(1) \begin{equation*} {Q}_j,{K}_j,{V}_j\kern0.5em \leftarrow \hat{X}\boldsymbol{W}_j^Q,\kern0.5em \hat{X}\boldsymbol{W}_j^K,\hat{X}\boldsymbol{W}_j^V \end{equation*}

(2) \begin{equation*} {{head}}_{{j}}={softmax}\left(\frac{{{Q}}_{{j}}\bullet{\left({{K}}_{{j}}\right)}^{{T}}}{\sqrt{{{d}}_{{k}}}}\right)\bullet{{V}}_{{j}} \end{equation*}

#### Hierarchy encoder

The second component of our model is the hierarchy encoder, which acts as an aggregation of information for the enzyme label space. The graph convolutional neural network GCN [34] has been widely utilized as structure encoder for aggregating node information. We combine the enzyme taxonomic hierarchy graph ${G}=({V},\overrightarrow{{E}},\overleftarrow{{E}})$ to design a three-layer hierarchy-GCN encoder to obtain fine-grained enzyme label hierarchy information.

We first use deductive method to align the enzyme sequence features of *S* with the label features of *G* to produce the node inputs *T* of GCN [30]. The feature matrix *S* is reshaped into *T* by the linear transformation:


(3) \begin{equation*} {T}={\boldsymbol{M}}_{{l}}{S}{\boldsymbol{M}}_{{c}} \end{equation*}

where ${\boldsymbol{M}}_{{l}}\in{\mathbb{R}}^{{C}\times{{d}}_{{k}}}$and ${\boldsymbol{M}}_{{c}}\in{\mathbb{R}}^{{h}\times{{d}}_{{t}}}$ are trainable weight matrices, and ${{d}}_{{t}}$ is the dimension for each label node of *G*.

In *G*, each directed edge represents a pair-wise label correlation feature. To formulate enzyme label correlations, given an enzyme label node *v_k_* in *G*, we describe the label hierarchy direction of *v_k_* with the following adjacent matrices:


(4) \begin{equation*} {\overset{\sim }{{A}}}_{{k}}^{\uparrow }={I}+{{A}}_{{\overrightarrow{{E}}}_{{k}}} \end{equation*}

(5) \begin{equation*} {\overset{\sim }{{A}}}_{{k}}^{\downarrow }={I}+{{A}}_{{\overleftarrow{{E}}}_{{k}}} \end{equation*}

where ${A}=\left\{{{a}}_{\mathbf{0},\mathbf{0}},{{a}}_{\mathbf{0},\mathbf{1}},\dots, {{a}}_{{C}-\mathbf{1},{C}-\mathbf{1}}\right\}$ is the adjacent matrix of *G*, ${I}\in{\mathbb{R}}^{{C}\times{C}}$ is identity matrix and *C* is the enzyme label number of *G*.${\overrightarrow{{E}}}_{{k}}=\left\{\left({{v}}_{{k}},{{v}}_{{j}}\right)\left|{{v}}_{{k}}\in{V},{{v}}_{{j}}\in{child}\left({{v}}_{{k}}\right)\right.\right\}$ and ${\overleftarrow{{E}}}_{{k}}=\left\{\left({{v}}_{{j}},{{v}}_{{k}}\right)\left|{{v}}_{{k}}\in{V},{{v}}_{{j}}\in{child}\left({{v}}_{{k}}\right)\right.\right\}$ are the top-down and bottom-up hierarchy paths of *v_k_* in *G*. In *A*, we can employ ${{a}}_{{k},{j}}=\mathbf{1}$ to obtain ${{A}}_{{\overrightarrow{{E}}}_{{k}}}$ and employ ${{a}}_{{j},{k}}=\mathbf{1}$to obtain ${{A}}_{{\overleftarrow{{E}}}_{{k}}}$.

Then we utilize each layer of our hierarchy-GCN encoder to aggregate the dataflows of the enzyme label structure within the top-down and bottom-up edges connecting *v_k_* in *G*. Formally, for the node inputs *T*, the first layer of GCN encodes the hidden state *g_k_* of the enzyme label node *v_k_* as follows:


(6) \begin{equation*} {{e}}_{{k}}^{\uparrow }={\hat{{D}}}^{-\frac{\mathbf{1}}{\mathbf{2}}}{\overset{\sim }{{A}}}_{{k}}^{\uparrow }{\hat{{D}}}^{-\frac{\mathbf{1}}{\mathbf{2}}}{T}{\boldsymbol{W}}^{\uparrow } \end{equation*}

(7) \begin{equation*} {{e}}_{{k}}^{\downarrow }={\hat{{D}}}^{-\frac{\mathbf{1}}{\mathbf{2}}}{\overset{\sim }{A}}_k^{\downarrow }{\hat{D}}^{-\frac{1}{2}}T\boldsymbol{W}^{\downarrow } \end{equation*}

(8) \begin{equation*} {e}_k^{bi}= Relu\left({e}_k^{\uparrow}\oplus{e}_k^{\downarrow}\right) \end{equation*}

(9) \begin{equation*} {g}_k=T+{e}_k^{bi} \end{equation*}

Here $\hat{D}\in{\mathbb{R}}^{C\times C}$ represents the normalized node degree matrix of *G*, $\boldsymbol{W}^{\uparrow },\boldsymbol{W}^{\downarrow}\in{\mathbb{R}}^{d_t\times{d}_g}$ are trainable weight matrices, $\oplus$ indicates the concatenation of matrices and *d_g_* is the output dimension of GCN layer. First, in order to model the enzyme label correlations of *v_k_*, we can separately use equations (6) and (7) to bidirectionally calculate the edge-wise transformation matrices ${e}_k^{\uparrow}\in{\mathbb{R}}^{d_t\times{d}_g}$ and ${e}_k^{\downarrow}\in{\mathbb{R}}^{d_t\times{d}_g}$for the edges connecting *v_k_* in bottom-up and top-down manners.

We next can use formula (8) to fuse ${e}_k^{\uparrow }$ with ${e}_k^{\downarrow }$ to obtain the bidirectional enzyme label correlation matrix ${e}_k^{bi}\in{\mathbb{R}}^{d_t\times{d}_g}$ of *v_k_*. Then we employ formula (9) to fuse the node inputs *T* with ${e}_k^{bi}$ to produce the final hidden state *g_k_* of *v_k_*.

Finally, the hidden state *g_k_* is fed into the next layer of GCN as the enzyme sequence feature vector. A similar process will be iteratively repeated until three layers of GCN have been computed. Once this repetition is completed, the output value of *g_k_* is mapped to the final prediction probability of *v_k_* for the given enzyme sequence *x* through a fully connected layer.

#### Loss function

Loss function is usually adopted to measure the difference between the real value and the predicted value. Cross-entropy loss function [35] is commonly used in multi-classification problems. Given a predicted enzyme label $\overline{Y}$ and its corresponding true label $Y$, we use the following cross-entropy loss function to optimize the distribution between real labels and the predicted labels:


(10) \begin{equation*} F=-\left[ Ylog\overline{Y}+\left(1-Y\right)\mathit{\log}\left(1-\overline{Y}\right)\right] \end{equation*}

Considering that the weight parameters of the fully connected layer are susceptible to the data imbalance of the enzyme hierarchy classes in GCN, we utilize the following recursive regularization term [36] to regularize the parameters of the final fully connected layer:


(11) \begin{equation*} \lambda \left(\omega \right)={\sum}_{v_i\in V}\sum \frac{1}{2}{\left\Vert \boldsymbol{w}_i-\boldsymbol{w}_j\right\Vert}^2 \end{equation*}

where the parameter set $\omega$ for the enzyme label node ${v}_i$ and its associated child node ${v}_j$ can be denoted as $\omega =\left\{\left(\boldsymbol{w}_i:\boldsymbol{w}_j\in \mathcal{L}\right)\right. \left. \left|{v}_i\in V,{v}_j\in child\left({v}_i\right)\right.\right\}$, *w_i_,* and *w_j_* are the parameters of the final fully connected layer $\mathcal{L}$ for ${v}_i$ and ${v}_j$ in *G*. Finally, we add the recursive regularization term formula (10) to the cross-entropy loss as the final loss function to optimize the model:


(12) \begin{equation*} J=F+ H\lambda \left(\omega \right) \end{equation*}

where *H* is the penalty parameter.

#### Model training

The complexity of model introduces a heightened risk of overfitting. We employ three methods to avoid overfitting in the predictions. The first approach is dropout [37]. The key of this technique lies in randomly dropping out a portion of neurons during training to prevent the network from relying on specific details, thereby reducing the risk of overfitting. The second approach involves dynamic adjustment of learning rates and early stopping for training. Specifically, during model training, we monitor the model’s performance on a validation set under a dynamic learning rate with fixed decay strategy. Training is halted when the performance no longer improves, thereby preventing overfitting. The third approach is to allocate different weights to different enzyme classes. By this way, we can ensure that the model performs well across different enzyme classes, avoiding overfitting in the predictions and mitigating the impact of data imbalance issue.

**Figure 3:**
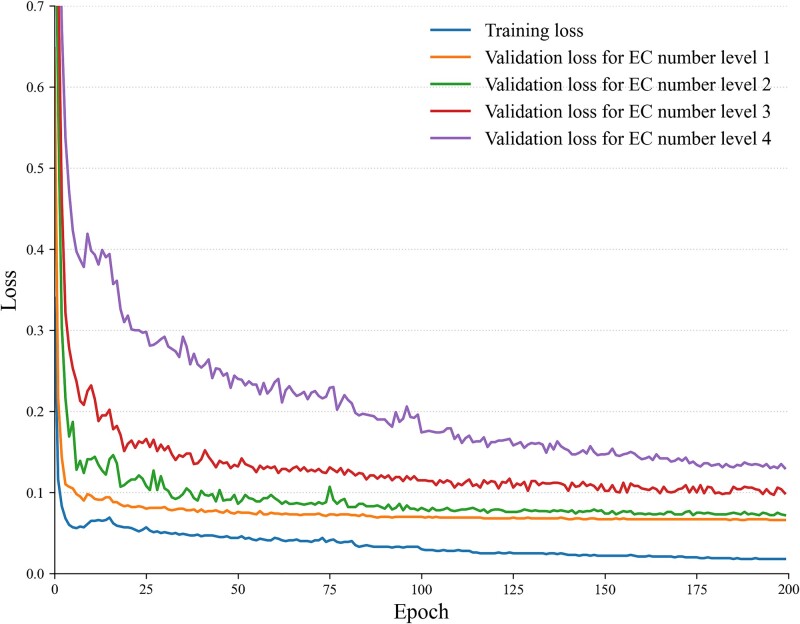
The training curves of GloEC model on the basic training dataset.

To develop our model, we train GloEC on the basic training dataset for 216 epochs using a Tesla T4 GPU. Each epoch takes approximately 1 hour to execute. The Adam optimizer [38] is chosen, with a batch size set to 256. Figure 3 illustrates the training curves of GloEC. In these curves, both the training loss and validation loss decrease as the number of training epochs increases, and training concludes when the validation set loss stops decreasing. Overall, the model demonstrates good convergence speed throughout the training process.

## Results and discussion

### Evaluation criteria

To evaluate the effectiveness of GloEC, we compared GloEC with three available state-of-the-art methods ProteInfer [19], DeepEC [14], and CLEAN [11] on three different benchmark datasets New-432, COFACTOR-237, and Isoenzyme. It is worth noting that DeepEC can only divide enzymes into six categories from EC1 to EC6 and, therefore, it cannot predict the EC7-related enzymes. To make fair comparisons, we removed the EC7-related enzymes from these three benchmark datasets and test DeepEC on the adjusted benchmark datasets, and tested other competing methods on the complete benchmark datasets.

The parameters of the comparative methods are the default values given by their articles [11, 14, 19]. To assess the competing methods, we use precision, recall, and macro-F1 scores, which are defined below in terms of false negatives (*FN*), false positives (*FP*), true negatives (*TN*), and true positives (*TP*), to evaluate the classifier’s performance:


(13) \begin{equation*} Precision=\frac{TP}{TP+ FP} \end{equation*}

(14) \begin{equation*} Recall=\frac{TP}{TP+ FN} \end{equation*}

(15) \begin{equation*} F1- score=\frac{2 TP}{2 TP+ FP+ FN} \end{equation*}

(16) \begin{equation*} Macro-F1=\frac{\sum_{i=1}^BF1-{score}_i}{B} \end{equation*}

Noted that, for each enzyme sequence dataset, Macro-F1 is the average of the F1-scores for each enzyme category and *B* represents the number of EC number categories for each enzyme EC level of the dataset [39].

### New-432 dataset

The New-432 dataset was not included in any model’s training, which ensures us to perform fair comparisons on different models. Table 2 shows the results of GloEC, ProteInfer, DeepEC, and CLEAN on the New-432 dataset.

**Table 2:** Performance comparison of GloEC, ProteInfer, DeepEC, and CLEAN on the New-432 dataset.

Enzyme EC levels	GloEC			ProteInfer			DeepEC			CLEAN		
	Macro-F1	Pre	Rec	Macro-F1	Pre	Rec	Macro-F1	Pre	Rec	Macro-F1	Pre	Rec
Level 1	**92.0**	**92.7**	**91.4**	71.4	69.2	75.0	68.7	65.6	75.8	87.4	87.0	88.2
Level 2	**84.3**	**85.6**	**84.1**	60.2	67.7	60.4	50.4	49.5	55.8	69.3	68.8	71.9
Level 3	**78.1**	**81.5**	**77.5**	44.7	48.9	43.4	39.6	42.1	43.5	63.7	66.9	64.3
Level 4	**53.7**	**55.9**	**54.1**	29.2	32.3	28.6	28.7	30.8	29.1	23.9	24.7	24.4

The best performers are highlighted in bold.

As shown in Table 2, GloEC performs better than other methods on almost all levels. For example, on level 4, GloEC reports a macro-F1 score of 53.7% while ProteInfer, DeepEC, and CLEAN scores 29.2, 28.7, and 23.9%, respectively. Similarly, on level 3, GloEC achieves a macro-F1 score of 78.1% as compared to 44.7, 39.6, and 63.7% achieved by ProteInfer, DeepEC, and CLEAN, respectively. The evaluation on the New-432 dataset indicates that GloEC is an effective method for predicting enzyme function.

### COFACTOR-237 dataset

In this experiment, we directly compared the performance of different methods in predicting the first-digit to fourth-digit of the enzyme using the benchmark dataset COFACTOR-237. COFACTOR-237 has been proved to be a tough dataset in the field of enzyme function prediction [28]. All samples of COFACTOR-237 have the latest enzyme annotation in UniPort database [40] (July 2023) and COFACTOR-237 has less than 80% sequence similarity to the GloEC basic training dataset. We manually input the 237 sequences contained in COFACTOR-237 into each comparative model and collect their prediction results.

As shown in Table 3, although DeepEC’s overall performance is superior to comparative methods for the first digit prediction, GloEC achieves better performance than other methods for the second-digit to fourth-digit prediction. On level 2, GloEC gives a macro-F1 score of 87.8% as compared to 76.3, 86.9, and 82.1% obtained by ProteInfer, DeepEC, and CLEAN, respectively. On level 3, GloEC reports a macro-F1 score of 80.8% as compared to 79.6, 77.1, and 75.6% achieved by ProteInfer, DeepEC, and CLEAN, respectively. Similarly, on level 4, GloEC improves macro-F1 score by at least 7% over the other models. These results demonstrate that GloEC has better generalization ability for cross-dataset validation, especially for deeper EC number prediction.

**Table 3:** Performance comparison of GloEC, ProteInfer, DeepEC, CLEAN, and COFACTOR on the COFACTOR-237 dataset.

Enzyme EC levels	GloEC			ProteInfer			DeepEC			CLEAN		
	Macro-F1	Pre	Rec	Macro-F1	Pre	Rec	Macro-F1	Pre	Rec	Macro-F1	Pre	Rec
Level 1	88.4	87.4	89.8	87.6	**94.8**	86.5	**92.2**	93.5	**91.3**	83.9	84.9	83.7
Level 2	**87.8**	**86.8**	**90.7**	76.3	79.9	75.9	86.9	86.7	87.7	82.1	81.6	85.3
Level 3	**80.8**	83.2	**81.2**	79.6	**83.6**	78.6	77.1	78.6	77.9	75.6	77.3	76.7
Level 4	**72.4**	**73.4**	**73.1**	65.4	65.6	65.2	63.9	64.5	63.7	62.6	63.1	63.3

The best performers are highlighted in bold.

### Isoenzyme dataset

Isoenzymes [41] are protein subtypes of enzymes that come from a single gene or family of genes and differ due to genetic differences, but these isomers usually perform the same function with different sequence length. It is thus a challenging task to correctly predict the function of isomers with different lengths. To further evaluate our method, we compared the performance of GloEC and other enzyme function prediction tools using the Isoenzyme dataset and the results are shown in Table 4.

**Table 4:** Performance comparison of GloEC, ProteInfer, DeepEC, and CLEAN on the Isoform dataset.

Enzyme EC levels	GloEC			ProteInfer			DeepEC			CLEAN		
	Macro-F1	Pre	Rec	Macro-F1	Pre	Rec	Macro-F1	Pre	Rec	Macro-F1	Pre	Rec
Level 1	**90.2**	91.2	**91.3**	89.9	**92.3**	87.9	83.6	84.0	83.4	87.3	85.4	90.1
Level 2	**87.6**	**87.8**	**86.2**	71.2	76.3	69.1	70.8	71.2	72.2	75.4	75.8	78.7
Level 3	**83.8**	**86.3**	**83.4**	65.0	69.1	63.1	65.8	67.0	66.4	76.4	77.8	78.7
Level 4	**75.5**	**76.6**	**74.9**	62.4	64.3	61.8	50.2	52.8	49.1	66.0	67.5	66.1

The best performers are highlighted in bold.

As can be seen in Table 4, GloEC outperforms other methods in terms of precision, recall and macro-F1 scores on almost all levels. Although the precision of ProteInfer is higher than other methods for the first level due to the data imbalance in the main class of the training dataset, GloEC obtains the best precision for the second to fourth levels and achieves the highest recall and macro-F1 scores for the first to fourth levels. The results of Table 4 demonstrate that GloEC can effectively predict the function of the isoforms.

### Carbohydrate esterase dataset

Below, we discuss the classification performance of GloEC on enzyme promiscuity. Carbohydrate-active enzymes (CAZymes) are a class of enzymes involved in carbohydrate metabolism in organisms [42]. Many CAZymes families such as the carbohydrate esterase family are built on sequence homology, which is expected to reflect similar three-dimensional structures [29]. However, minor differences in these sequences may lead to enzymes performing more than one function, albeit with less specificity. As of now, according to the classification in the CAZy database (Carbohydrate-Active enZYmes Database), carbohydrate esterase is divided into 20 different families (CE1–CE20) [43]. We curate the carbohydrate esterase family from the TrEMBL database [44], which included 354 enzyme samples with 7 different EC numbers. Then we test the performance of GloEC, ProteInfer, DeepEC, and CLEAN on predicting EC numbers for carbohydrate esterase family and the results are shown in Table 5. As can be seen in Table 5, GloEC exhibits the best macro-F1 scores (49.8, 27.0, 24.0, and 13.9%) across various levels. The classification results on the carbohydrate esterase family demonstrate that GloEC is able to classify the enzyme family built on sequence homology.

**Table 5:** The performance of GloEC, ProteInfer, DeepEC, and CLEAN on predicting EC numbers for carbohydrate esterase family.

Enzyme EC levels	GloEC			ProteInfer			DeepEC			CLEAN		
	Macro-F1	Pre	Rec	Macro-F1	Pre	Rec	Macro-F1	Pre	Rec	Macro-F1	Pre	Rec
Level 1	**49.8**	**50.0**	**67.7**	27.4	25.0	24.5	24.1	25.0	23.2	31.9	3.33	30.6
Level 2	**27.0**	**28.1**	**45.9**	17.4	16.4	27.9	12.8	14.5	11.9	26.3	27.3	26.4
Level 3	**24.0**	**25.2**	**41.4**	14.1	15.0	13.4	16.7	18.7	15.6	20.6	21.1	20.2
Level 4	**13.9**	**30.5**	**32.3**	7.91	10.8	7.03	7.16	10.7	6.66	13.0	13.6	12.6

The best performers are highlighted in bold.

The results of Table 5 show that, although GloEC can effectively classify most enzymes of carbohydrate esterase family, GloEC is still struggling with distinguishing the enzyme sequences with low specificity. For example, in UniProt database [40], glucuronoyl esterase (EC 3.1.1.117) is classified to the carbohydrate esterase family. Although GloEC predicts glucuronoyl esterase as a member of the carbohydrate esterase family (EC 3.1.1.-) since the sequences of glucuronoyl esterase are similar to the members of the carbohydrate esterase family, GloEC does not correctly identify the fourth EC number of glucuronoyl esterase. The minor differences in enzyme sequences with low specificity makes it difficult for the model to learn effective features, leading GloEC to tend towards conservative results during inference.

### Comparison of computing resource

**Figure 4:**
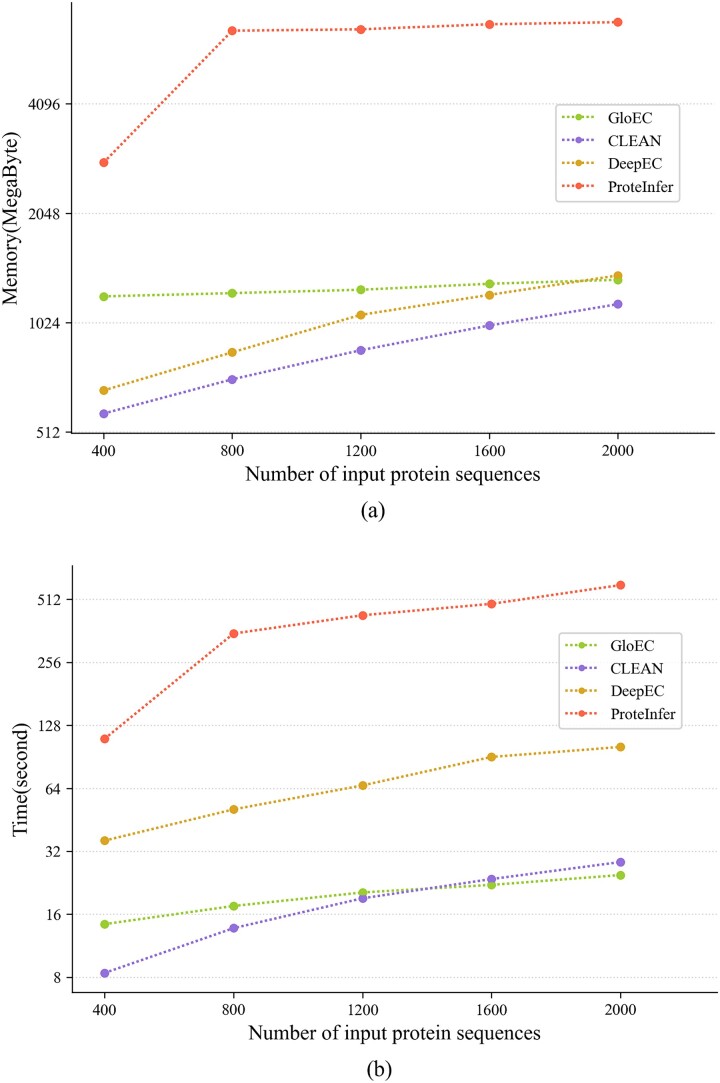
(a) The memory usage for different methods in annotating enzyme sequences. (b) The time usage for different methods in annotating enzyme sequences.

In order to learn the runtime complexity of different models on large dataset, we randomly select 400, 800, 1200, 1600, and 2000 enzyme sequences (see the data shared in https://github.com/hyr0771/GloEC/tree/master/02.Datasets/Different_sizes) from the TrEMBL database [44] and compare the memory and time usage of GloEC and three other tools in predicting EC numbers for these sequences. As shown in Fig. 4(a), among these four prediction tools, ProteInfer exhibits significantly higher memory requirements and time consumption compared to other tools. DeepEC and CLEAN have approximately only one-fifth of ProteInfer’s memory requirements. GloEC has moderate and stable memory requirements, with a smaller increase in memory demand as the dataset size grows. As we can see in Fig. 4(b), both GloEC and CLEAN are the fastest EC prediction tools, with a significant advantage in time consumption compared to DeepEC (<2 times) and ProteInfer (<10 times).

### The impact of limited samples

Data imbalance is a common issue, for example, in the basic training dataset, some EC numbers have over 1000 samples available for model learning, while some other rare EC numbers have only 10 samples. Severe data imbalance can result in model exhibiting prediction bias towards categories with more samples. In order to learn the model performance on classifying rare EC numbers, we curate a validation dataset consisting of enzymes associated with rare EC numbers from Swiss-port database [6]. This dataset comprises over 3000 enzyme samples, covering more than 1000 unique EC numbers, each EC number appearing no more than five times in enzyme samples (see the data shared in https://github.com/hyr0771/GloEC/tree/master/02.Datasets/Limited_Samples). Table 6 presents the performance of GloEC and other models on this dataset. As shown in Table 6, GloEC demonstrates the best prediction performance, indicating that it can correctly predict the majority of rare enzyme categories and showcase superior generalization compared to other models.

**Table 6:** Performance comparison of GloEC, ProteInfer, DeepEC, and CLEAN on the dataset with rare EC numbers.

Enzyme EC levels	GloEC			ProteInfer			DeepEC			CLEAN		
	Macro-F1	Pre	Rec	Macro-F1	Pre	Rec	Macro-F1	Pre	Rec	Macro-F1	Pre	Rec
Level 1	**98.6**	**98.5**	**98.6**	92.5	97.1	90.3	93.8	94.4	93.2	96.0	96.7	95.2
Level 2	**93.1**	**92.9**	**93.4**	75.4	80.4	74.9	82.6	85.1	83.6	86.9	87.0	88.5
Level 3	**90.4**	**90.8**	**90.4**	67.7	71.5	66.1	79.4	82.5	79.6	85.8	86.7	86.5
Level 4	**80.9**	**81.4**	**81.7**	72.9	74.0	72.8	73.9	74.5	74.3	74.5	74.3	76.2

The best performers are highlighted in bold.

### Ablation experiment

In GloEC, each layer of GCN is used to encode the enzyme label structure information. Generally speaking, the more encoding layers are used, the better enzyme label structure information could be aggregated [45]. In order to evaluate the effectiveness of the enzyme label structure information employed in GloEC, we construct five different GloEC models based on the number of GCN layers, namely GloEC-0, GloEC-1, GloEC-2, GloEC-3, and GloEC-4, which represent different GloEC modes using 0, 1, 2, 3, and 4 layers of GCN, respectively. Table 7 shows the macro-F1 score, precision, and recall of different GloEC models obtained from a 10-fold cross-validation experiment on the basic training dataset.

**Table 7:** Performance comparison of GloEC-0, GloEC-1, GloEC-2, GloEC-3, and GloEC-4 on the basic training dataset.

Enzyme EC levels	GloEC-0			GloEC-1			GloEC-2			GloEC-3			GloEC-4		
	Macro-F1	Pre	Rec	Macro-F1	Pre	Rec	Macro-F1	Pre	Rec	Macro-F1	Pre	Rec	Macro-F1	Pre	Rec
Level 1	99.1	99.1	98.4	99.1	**99.2**	98.7	**99.2**	**99.2**	**99.0**	**99.2**	**99.2**	98.8	99.1	99.1	99.0
Level 2	95.1	95.2	94.0	95.8	94.9	94.4	**97.4**	97.2	**97.8**	96.9	**97.6**	96.3	95.6	95.5	93.9
Level 3	82.1	82.7	78.9	91.9	90.7	88.7	**92.5**	**91.4**	**93.5**	92.4	90.9	92.7	89.2	87.4	89.4
Level 4	53.3	52.0	56.4	73.5	72.5	70.9	74.8	75.2	73.4	**75.7**	**76.7**	**74.1**	72.1	73.1	70.8

The best performers are highlighted in bold.

As can be seen in Table 7, in the prediction of four levels for enzyme EC number, all performance metrics of GloEC-0 are lower than those of other models. Especially at the fourth level, GloEC-0 has a macro-F1 score of 53.3%, while the macro-F1 scores of GloEC-1 to GloEC-4 are 73.5, 74.8, 75.7, and 72.1%, respectively. Considering that GloEC-0 does not encode enzyme label information, these results indicate that the performance of enzyme function prediction can be improved by incorporating the label structure information of enzyme into model training.

In Table 7 we can see that GloEC-2 outperforms GloEC-1 and GloEC-0 in predicting all four levels. This implies that increasing encoding layers can enhance the aggregation of enzyme label information so as to further improve the prediction performance. However, except for levels 1 and 4, the macro-F1 scores of GloEC-2 are higher than those of GloEC-3 for levels 2 and 3, and the performance metrics of GloEC-4 are lower than those of GloEC-3 across all four levels. The rationality behind is that the aggregation for enzyme label information relies on its neighborhood labels’ information and can only be enhanced by adding encoding layers based on the available neighborhood label information. The results in Table 7 suggest that three-layer GCN is the upper limit for aggregating available neighborhood label information of enzyme to infer 4-level EC number. However, people still can try more GCN layers in GloEC to infer label classification task with higher label level.

On the other hand, we also evaluated the effectiveness of hierarchy encoder and recursive regularization used in GloEC. We construct two different GloEC variants, namely GloEC-GCN and GloEC-Sin. GloEC-GCN is the variant that the recursive regularization is removed from GloEC. GloEC-Sin the variant that recursive regularization and our proposed hierarchy encoder are removed from GloEC. We perform a 10-fold cross-validation experiment for these two different GloEC variants on the basic training dataset. Table 8 shows the macro-F1 scores, precision, and recall for different variants on different levels of EC numbers.

**Table 8:** Performance comparison of GloEC, GloEC-GCN, and GloEC-Sin on the basic training dataset.

Enzyme EC levels	GloEC			GloEC-GCN			GloEC-Sin		
	Macro-F1	Pre	Rec	Macro-F1	Pre	Rec	Macro-F1	Pre	Rec
Level 1	**99.2**	**99.2**	98.8	99.1	99.1	**99.0**	99.0	99.1	98.7
Level 2	**96.9**	**97.6**	96.3	96.1	94.6	**96.5**	92.1	90.4	92.0
Level 3	**92.4**	**90.9**	**92.7**	91.0	90.4	89.4	80.5	78.4	80.1
Level 4	**75.7**	**76.7**	**74.1**	72.4	75.0	71.7	63.2	60.6	65.6

The best performers are highlighted in bold.

As shown in Table 8, GloEC-GCN achieves better performance than GloEC-Sin for the first to fourth levels. Especially on the third and fourth levels, GloEC-GCN has the macro-F1 scores of 72.4 and 91%, which are at least a 9% improvement over GloEC-Sin, respectively. This result demonstrates the proposed hierarchy encoder could play a critical role in predicting enzyme function. In addition, as illustrated in Table 8, GloEC obtains higher macro-F1 score than those of GloEC-GCN for almost all levels. This demonstrates that recursive regularization could be an effective complementary to our proposed architecture.

### Interpretability analysis

**Figure 5:**
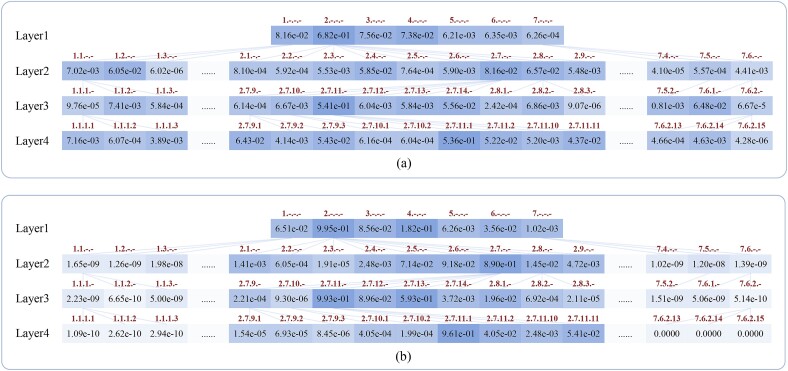
(a) The initial weight of each enzyme label in the enzyme graph for the input sequence of threonine-protein kinase. (b) The weights of the enzyme labels in the graph after updating by the hierarchy-GCN encoder.

In order to learn the interpretability of the predictions of our model, we use the sequence of threonine-protein kinase (EC 2.7.11.1) [46] as a test input for GloEC and trace the enzyme label weights of the enzyme graph in Fig. 5. In Uniport database [40], the EC number of threonine-protein kinase is classified to 2.7.11.1. In this example, GloEC first computes the initial weight of each enzyme label in the enzyme graph for threonine-protein kinase based on enzyme sequence features, and the results are shown in Fig. 5(a). Then based on the hierarchical dependency of enzyme labels, the hierarchy-GCN encoder bidirectionally updates the initial weights of enzyme labels with enzyme sequence features within the same enzyme group. After updating weights by the abovementioned way, as can be seen in Fig. 5(b), the weights of enzyme label nodes in each enzyme group update towards to agree due to their hierarchical connectivity and the weight of the enzyme label 2.7.11.1 is updated to the maximum value in the graph. Finally, GloEC correctly identifies the enzyme label 2.7.11.1 with maximal weight as the EC number of threonine-protein kinase.

In this example, we can see that, bidirectionally updating enzyme label weights based on hierarchical dependency of enzyme labels could be an effective method to capture fine-grained label-correlation hierarchy information of enzymes to make prediction.

### Case study

In this section we applied GloEC to predict enzyme function in practical applications. The classification of two specific isoenzymes will be discussed below. Glutamine occupies a central position in cellular metabolism: Glutamine is not only a component of most proteins, but also a source of nitrogen in biosynthetic pathways [47]. Thus, the enzyme that catalyzes glutamine synthesis (glutamine synthetase, EC 6.3.1.2) plays a key role in cell metabolism. There are three isoforms of glutamine synthetase II in *Drosophila melanogaster*, all of which have enzyme activity.

To verify GloEC’s ability for identifying different isoforms of glutamine synthetase II, we collected the sequences of these three isoforms of glutamine synthetase II from Swiss-Port, among which the sequences of the first subtype was included in the training set, so we put the sequences of the remaining isoforms glutamine synthetase II-2 and glutamine synthetase II-3 into our model for prediction. Finally, GloEC identified that both of glutamine synthetase II-2 and glutamine synthetase II-3 belong to glutamine synthetase, which is consistent with the experimental results.

The Cystic fibrosis transmembrane conductance regulatory (CFTR) is a channel conductance controlling ATPase (EC 5.6.1.6) and its absence in human could lead to cystic fibrosis [48]. Swiss-Port recorded two other isoforms CFTR-2 and CFTR-3 of CFTR, whose sequence lengths are less than half that of the ‘canonical’ isoform (1476 amino acids versus 576 and 600 amino acids). Despite being much short in length, the isoforms CFTR-2 and CFTR-3 do not lose their function.

To verify our GloEC’s capability of predicting subtypes’ functionality, we obtained the sequence of CFTR-2 and CFTR-3 from Swiss-Port and fed them into our model. Particularly, GloEC successfully predicted their functions, while none of the other three methods, CLEAN, ProteiInfer, and DeepEC, gave correct predictions. This indicates that GloEC is able to capture the function of the isoforms of CFTR, even though their sequences are very different from the ‘canonical’ sequence.

**Figure 6:**
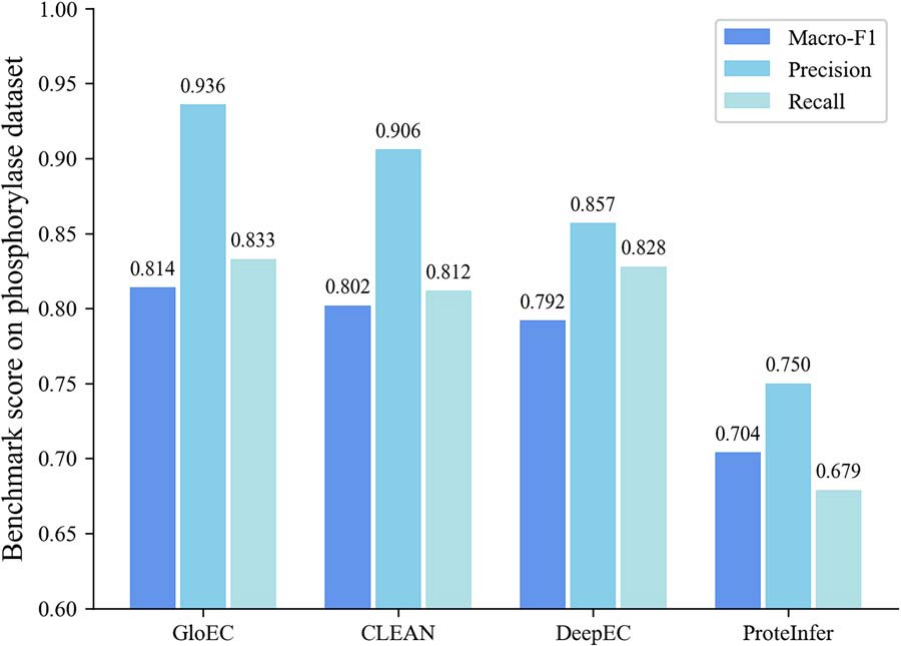
The performance of GloEC, ProteInfer, DeepEC, and CLEAN on predicting the fourth-level EC numbers of phosphorylases.

Phosphorylases play a crucial role in glycogen metabolism, particularly in muscle and liver tissues [49]. Swiss-Prot [6] has identified over 10 types of phosphorylases, such as glycogen phosphorylase, purine-nucleoside phosphorylase, methylthioadenosine phosphorylase, and adenosylhomocysteine nucleosidase. Each type of phosphorylases exhibits analogous catalytic capabilities in catalyzing phosphorolysis reactions [50]. We collect an enzyme set of phosphorylases from Swiss-Prot database [6], comprising 37 sequence samples and 6 types of phosphorylases. For these phosphorylases, GloEC and other competitive methods can correctly identify the first three levels of EC numbers and, therefore, we compare the performance of all methods on identifying the fourth-level EC number, and the results are shown in Fig. 6. As can be seen in Fig. 6, compared to other tools, GloEC achieves the best precision (93.6%), macro-F1 score (81.4%), and recall (83.3%) at the fourth-level EC number. The EC number identification results for phosphorylases suggest that GloEC can effectively distinguish the function of enzymes that exhibit comparable enzymatic activities.

### Limitations

Despite the effectiveness of GloEC in identifying enzyme function, there certainly remains room for improvement. Firstly, there is a significant need to increase the coverage of rare EC numbers in the basic training dataset. As observed in this study, the protein sequence coverage for each EC number varies greatly, with 910 out of 1643 EC numbers having fewer than 10 protein sequence samples covered. This is also why GloEC did not correctly assign substrate class numbers for glucuronoyl esterase (EC 3.1.1.117). Addressing such data imbalance problem has the potential to enhance the predictive performance of GloEC, particularly in terms of precision and coverage for predicting EC numbers.

Secondly, GloEC focuses on classifying enzymes into a single EC number. However, multifunctional enzymes may exhibit different catalytic activities in different contexts. For example, Fatty Acid Synthase (FAS) is a multifunctional enzyme complex responsible for catalyzing multiple steps in fatty acid biosynthesis. FAS can act as a β-ketoacyl synthase (EC 2.3.1.41), catalyzing the condensation of acetyl-ACP and malonyl-ACP to form β-ketoacyl-ACP. Meanwhile, FAS also can act as a β-ketoacyl-ACP reductase (EC 1.1.1.100), reducing β-ketoacyl-ACP to β-hydroxyacyl-ACP [51]. We use FAS for testing, but GloEC only provides a prediction for one EC number (EC 2.3.1.41) and does not give another EC number (EC 1.1.1.100), which is inaccurate in practical applications. In the future, we plan to assign enzyme labels with predicted probabilities above a certain threshold to identify whether enzymes are multifunctional or monofunctional, helping us to expand the model to predict multiple functions of enzymes.

Lastly, the results of this work are primarily validated by computational predictions based on previous benchmark data, without experimental validation for new predictions. The predicting results should undergo rigorous and thorough analysis and in-depth study before proceeding with experimental implementations. Such implementations could involve verifying the model’s predictions *in vitro* in the future.

## Conclusion

In this article, we propose a novel hierarchical-aware deep-learning model GloEC for enzyme function prediction. GloEC concentrates on integrating the directed enzyme graph to globally build a hierarchy-GCN encoder to model and extract the hierarchy information of the enzyme labels. Furthermore, the bidirectional calculation of the hierarchy-GCN encoder allows it to comprehensively learn the label correlation information of enzymes in both bottom-up and top-down directions, enabling us to accurately utilize the hierarchy structural information of enzyme label for annotating enzyme function.

The effectiveness of GloEC was validated by comparative experiments on gold standard datasets. The results demonstrate that GloEC performs better than the existing methods in terms of precision, recall, and macro-F1 scores. Case studies demonstrate that GloEC can accurately identify the function of isozymes even if they contain a big difference in sequence length. GloEC thus could be an applicable tool for predicting the catalytic function of enzymes, potentially paving the way for the identification of cost-effective and better enzymes for commercial applications.

**Key Points**

- A higher enzyme EC level relies on the lower enzyme EC level.
- GloEC models the hierarchical dependence of enzyme labels.
- The hierarchy-GCN encoder is bidirectionally computed.
